# Calcium Channels in Postnatal Development of Rat Pancreatic Beta Cells and Their Role in Insulin Secretion

**DOI:** 10.3389/fendo.2018.00040

**Published:** 2018-03-05

**Authors:** Neivys García-Delgado, Myrian Velasco, Carmen Sánchez-Soto, Carlos Manlio Díaz-García, Marcia Hiriart

**Affiliations:** ^1^Neuroscience Division, Cognitive Neuroscience Department, Instituto de Fisiología Celular, Universidad Nacional Autónoma de México UNAM, Mexico City, Mexico; ^2^Posgrado en Ciencias Biomédicas UNAM, Mexico City, Mexico

**Keywords:** low-voltage-activated and high-voltage-activated calcium channels, postnatal development, insulin secretion, reverse hemolytic plaque assay, beta-cell maturation

## Abstract

Pancreatic beta cells during the first month of development acquire functional maturity, allowing them to respond to variations in extracellular glucose concentration by secreting insulin. Changes in ionic channel activity are important for this maturation. Within the voltage-gated calcium channels (VGCC), the most studied channels are high-voltage-activated (HVA), principally L-type; while low-voltage-activated (LVA) channels have been poorly studied in native beta cells. We analyzed the changes in the expression and activity of VGCC during the postnatal development in rat beta cells. We observed that the percentage of detection of T-type current increased with the stage of development. T-type calcium current density in adult cells was higher than in neonatal and P20 beta cells. Mean HVA current density also increased with age. Calcium current behavior in P20 beta cells was heterogeneous; almost half of the cells had HVA current densities higher than the adult cells, and this was independent of the presence of T-type current. We detected the presence of α1G, α1H, and α1I subunits of LVA channels at all ages. The Cav 3.1 subunit (α1G) was the most expressed. T-type channel blockers mibefradil and TTA-A_2_ significantly inhibited insulin secretion at 5.6 mM glucose, which suggests a physiological role for T-type channels at basal glucose conditions. Both, nifedipine and TTA-A_2_, drastically decreased the beta-cell subpopulation that secretes more insulin, in both basal and stimulating glucose conditions. We conclude that changes in expression and activity of VGCC during the development play an important role in physiological maturation of beta cells.

## Introduction

Pancreatic beta cells acquire functional maturity during the first month of life, allowing them to present a robust insulin secretion in response to increases in the extracellular glucose concentration. Beta cells of adult animals are mature and detect changes in the extracellular glucose level; presenting a biphasic secretion, consisting of a first fast phase and a second one sustained (1). In contrast, fetal and neonatal cells are considered immature, secrete less insulin than adults and do not discriminate between different glucose levels. In fact, fetal mammals depend on their mother to regulate glucose homeostasis (2).

At birth, animals become independent, and this is a driving force for beta cells to mature. Another crucial development stage is the weaning period (20 days in rats, P20), where we have observed a physiological insulin resistance (3). This is a critical postnatal window due to changes in animal food, a shift from a high-fat diet to high-carbohydrate diet, which may lead to changes in the cellular response machinery, including receptors, enzymes, and ion channels activity.

We have also previously demonstrated that barium current and the expression levels of α1C, D, and G subunits of voltage-gated calcium channels (VGCC) in neonatal cells is lower than in adult cells and these changes relate to insulin secretion (4).

The studies about development using experimental animals should take into consideration the differences in anatomy, physiology, development, and biological process between rats and humans (5). It is not possible to give an exact equivalence. However, one study revealed that on average 16.7 rat days are equivalent to one human year (6). But, most studies consider that the relative ages are distinct depending upon the phase of life (5, 7). Taking into account, the developmental stages of rat life, the equivalences are the following, 42.4 rat days ~ one human year (weaning period) and 10.5 rat days ~ one human year (adulthood). It is also important to understand this correlation type because the average weaning age for laboratory rats is 3 weeks, but for humans is approximately 180 days. Furthermore, rats develop rapidly and become sexually mature at about 6 weeks, while humans grow slowly, and they reach puberty about 11–12 years (7).

Glucose-stimulated insulin secretion (GSIS) in adults is coupled to the electrical activity of the beta cell (8, 9). GSIS is a Ca^2+^-dependent process where the increase in ATP/ADP ratio induced by glucose metabolism close a K_ATP_ channel, depolarizes the cell membrane and activates sodium and calcium voltage-dependent channels. Ca^2+^ entry through different types of VGCC triggers the exocytosis of the insulin contained in secretion granules (10, 11).

Voltage-gated calcium channels are protein complexes formed by different subunits. The α1 subunits form the ion-conducting pore and channels are classified according to the primary structure of the α1 subunits in: Cav 1.1–1.4 (L-type), Cav 2.1 (P/Q-type), Cav 2.2 (N-type), Cav 2.3 (R-type), and Cav 3.1–3.3 (T-type) (12). VGCC are also classified according to biophysical properties. High-voltage-activated (HAV) Ca^2+^ channels (HVA, Cav1 and Cav2) open responding to strong depolarizations, have high conductance, conduct Ba^2+^ better than Ca^2+^ and do not inactivate during long-lasting voltage pulses. Between HVA channels, L-type channels are considered the most important Ca^2+^ entry pathway controlling insulin exocytosis in rodents and beta-cell lines (13–15).

On the other hand, the low-voltage-activated (LVA) Ca^2+^ channels (LVA or T-type, Cav3) have been poorly studied in native beta cells. These channels have unique biophysical and structural properties. They open at membrane potentials more negative and have smaller conductance and fast activation and inactivation kinetics, than HVA channels. Structurally, T-type channels are formed exclusively by the α1 subunit and they do not have regulatory subunits (16). T-Type channels are present in the beta cells of most of the species studied (15) and they are necessary for an adequate insulin secretion (17, 18), as well as for triggering beta-cell death in response to cytokines (19).

Furthermore, a recent work of our group revealed that transcriptome changes are involved in the beta-cell maturation, by microarray analysis (20). We showed a differential expression in P20 and adult cells in a variety of gene sets including those involved in excitability. Notably, expression of Cacna1h gene encoding for the α1H subunit of T-type calcium channels was lower in P20 than adult cells.

To better understand the postnatal beta-cell maturation, in this study we analyzed possible changes in the electrophysiological properties of LVA and HVA Ca^2+^ currents, as well as the expression of all T-type Ca^2+^ channels during different stages of postnatal development of rats. We also analyzed the relevance of both current types for insulin secretion process.

## Materials and Methods

### Chemical and Reagents

Reagents were obtained from the following sources: collagenase P, bovine serum albumin (BSA), Ficoll 400, trypan blue, 4-(2-hydroxyethyl)-1 piperazineethanesulfonic acid (HEPES), poly-L-lysine, *Staphylococcus aureus* protein A, trypsin, insulin, guinea-pig complement, nickel chloride (II), and all salts of electrophysiological recordings from Sigma-Aldrich (St. Louis, MO, USA); mibefradil, nifedipine, anti-VGCC rabbit polyclonal IgG from Alomone Labs (Jerusalem, Israel); FITC-conjugated anti-guinea pig, and Alexa Fluor 647-conjugated anti-rabbit IgG from Jackson ImmunoResearch (West Grove, USA). Tetrodotoxin from Calbiochem (La Jolla, CA, USA); TTA-A_2_ kindly donated by Merck (West Point, PA, USA) to Dr. Juan Carlos Gomora; tissue culture dishes from Corning (Corning, NY, USA); Hanks’ balanced salt solution, RPMI-1640 salts, and penicillin–streptomycin solution, fetal bovine serum, and L-glutamine solution from Thermo Fisher Scientific Inc. (Massachusetts, USA).

### Experimental Animals

All methods used in this study were approved by The Animal Care Committee of the Instituto de Fisiología Celular, Universidad Nacional Autónoma de México. Animal care was performed according to the International Guiding Principles for Biomedical Research Involving Animals, Council for International Organizations of Medical Sciences, 2010. Neonatal (1-day old), 20 days, and young adult (250–280 g) male Wistar rats were obtained from the local animal facility, maintained on a 14:10-h light–dark cycle (0600–2000), and allowed free access to standard laboratory rat diet and tap water.

### Culture of Pancreatic Beta Cells

On the day of the experiments, animals were anesthetized with intraperitoneal sodium pentobarbital (40 mg/kg), and after pancreas dissection, animals were killed by cervical dislocation. Pancreatic beta cells were obtained from neonatal (1-day old), 20 days, and young adult (250–280 g) male rats following a previously described procedure (3). Briefly, pancreatic islets were isolated and separated from the acinar tissue by collagenase digestion (0.5 mg/mL collagenase P) in Hank’s balanced salt solution, and a Ficoll (Sigma) gradient centrifugation; clean islets were then handpicked. Dissociation of the cells was achieved by incubating islets in a shaker bath for 5 min at 37°C in Ca^2+^-free Spinner solution, with 15.6 mmol/L glucose, 0.5% BSA, and 0.01% trypsin, followed by mechanical disruption. Before experiments, all single cells were cultured for 1 day in RPMI-1640 (11.6 mM glucose) supplemented with 10% fetal calf serum, 200 µM L-glutamine, 200 U/mL penicillin G, 200 µg/mL streptomycin, and 0.5 µg/mL amphotericin B allowing them to recover from dissociation. This glucose concentration in the culture medium was used because it has proven to maintain their function and survival.

### Electrophysiological Recordings

The whole-cell configuration of the patch-clamp technique (21) was used to record macroscopic voltage-gated Ca^2+^ currents, using Ca^2+^ as the charge carrier. Experiments were done at 22°C. The Axopatch 200 A amplifier (Axon Instruments, Foster City, CA, USA), was used. Patch electrodes with a tip resistance of 2–4 MΩ were pulled from capillary tubes KIMAX-51 (Kimble Glass, Vineland, NJ, USA). Electrode tips were coated with dental wax. The external solution consisted of (mmol/L): 130 NaCl, 5 KCl, 10 HEPES, 2 MgCl_2_, 5 CaCl_2_, and 10 Glucose, pH 7.3. The internal solution contained (mmol/L): 120 CsAsp, 10 CsCl, 5 CsF, 10 HEPES, 2.5 BAPTA, and 2 ATP, pH 7.3. Na^+^ current was blocked by the addition 100 nmol/L of TTX to the external solution. The capacity transient of the pipette was canceled before the cell membrane was ruptured, and total cell capacitance was determined by digital integration of capacitive transients with +10 mV pulses, from a holding potential of −80 mV. Cell capacitive transients were canceled, and using the internal voltage-clamp circuitry compensated series resistance. Remaining linear capacity transients and leakage currents were subtracted by a P/2 on line procedure.

We analyzed macroscopic calcium currents of beta cells with capacitances between 4.5 and 11 pF. It is possible to have a small percentage of other cell types.

The protocols used for the analysis of the calcium currents (ICa^2+^) consisted of a voltage ramp from −80 to +60 mV, 500 ms duration, and 0.5 mV/ms slope and depolarizing test pulses of 40 ms duration, from −60 to +40 in 5 mV increments, from a holding potential of −80 mV. ICa^2+^ current activation curves were obtained by converting the peak current values to conductance.

$$G{\text{ ICa}}^{2+}={\text{ICa}}^{2+}/(\text{Vm}-{\text{ErCa}}^{2+})$$

where ICa^2+^ is the peak current value, Vm is the command pulse potential, and ErCa^2+^ is the apparent reversal potential, obtained by extrapolation of the I–V relationships, which in most cases was ~ +40 mV.

Conductance values were normalized and fitted to the Boltzmann relation:

$$G/G_{\text{max}}=\{1+\text{exp}[-(V-{\text{Va}}_{1/2})/\text{ka}\}-1$$

where *G* is the ICa^2+^ peak conductance, *G*_max_ is the maximal ICa^2+^ conductance, Va_1/2_ is the midpoint of the activation curve, and ka is the activation steepness factor.

The specific LVA and HVA calcium channel blockers: nifedipine (5 µM), mibefradil (1 and 10 µM), TTA-A_2_ (10 and 50 µM), or nickel chloride (50 µM) were applied with the aid of a Picospritzer device (General Valve; Fairfield, NJ, USA).

### Double Immunostaining of Insulin and α1 Subunits of Low-Voltage-Gated Ca^2+^ Channels

Isolated islet cells were fixed with 2% paraformaldehyde in PBS for 30 min at room temperature, washed, and placed in a blocking solution containing 2% BSA (w/v) and 0.1% Triton X (v/v) for 15 min at room temperature. Samples were incubated in a humid chamber overnight at 4°C with primary antibodies raised against α1G, α1H, and α1I subunits of LVA channels (anti-VGCC rabbit polyclonal IgG; Alomone Labs; Jerusalem, Israel); at 1:100 dilution. The next day, samples were washed and incubated overnight at 4°C with the primary guinea-pig antibody anti-rat insulin (1:5,000 dilution). After a washing with PBS, samples were incubated for 2 h at room temperature with the secondary antibody FITC-conjugated goat anti-rabbit IgG (1:100 dilution) (Jackson Immuno Research; Pennsylvania, USA) and finally incubated for 1 h with the secondary antibody Alexa Fluor 647-conjugated goat anti-guinea-pig IgG (1:100 dilution) (Jackson Immuno Research; Pennsylvania, USA). Samples were incubated for 5 min with DAPI for visualizing nuclei and were mounted with medium containing 15 mM of NaN3 (DAKO). Negative controls were cells treated with the secondary antibodies alone.

#### Fluorescence Imaging of Cultured Pancreatic Cells and Single Cell Fluorescence Quantification

For imaging of specific immunoreactivity, cultured cells were viewed under epifluorescence microscopy using a Olympus IX71 inverted microscope equipped with a mercury lamp and a filter set appropriate for Alexa Fluor 647 (excitation 651 nm, emission 667 nm) and FITC (excitation 488 nm, emission 522 nm) and fluorescence images of each field were obtained separately. Samples were examined using an objective 20× and an oil immersion fluorescence objective 60×. Images were acquired with a Q Imagin digital camera, Image-Pro3DS 6.0 software, and stored in TIFF image format. Image analysis was performed with Image J 1.36 (Wayne Rasband; National Institutes of Health, USA). For each coverslip/condition, we determined the percentage of beta cells expressing the different subunits of LVA channels. Finally, for each pancreatic beta cell in the field, the cytoplasmic (non-nuclear) area was defined and the mean fluorescence intensity was measured from this area of interest in about 150–200 cells in separate fields per each condition. Background (control negative cells)-corrected fluorescence intensities (specific fluorescence) were calculated. Data were from four independent cultures (four rats/culture).

### Reverse Hemolytic Plaque Assay (RHPA)

To identify insulin-secreting cells and to measure insulin secretion by single cells, we used the RHPA (22) used previously in many analysis for pancreatic beta cells (23). Briefly, after 1 day in culture, 100,000 isolated islet cells were detached from culture dishes, and equal volumes of islet cells were mixed with *S. aureus* protein A-coated sheep erythrocytes, introduced to Cunningham chambers previously treated with poly-L-lysine to promote cell attachment and incubated for 1 h at 37°C and 5% CO_2_. Experiments were carried out at 5.6 or 15.6 mM glucose with or without each calcium channel blocker in the presence of primary guinea-pig insulin antiserum (1:40) (Strategic Biosolution, Newark, DE, USA), and then incubated for 1 h with guinea-pig complement (1:30). Insulin released was revealed by the presence of hemolytic plaques around the secreting cells which result from the complement-mediated lysis of erythrocytes bearing insulin–anti-insulin complexes bound to protein A. Each coverslip was scanned with a Leica Micro Dissection System 6000 (version 6.4.1.2887) coupled to a Hitachi HV-D20 camera. The percentage of insulin-secreting cells capable of forming immunoplaques and the immunoplaque area were estimated and the overall secretory activity of beta cells under a given experimental condition was expressed as a secretion index. This index was calculated by multiplying the average immunoplaque area times the percentage of plaque-forming cells (24). At least 100 cells were counted per duplicate in each experimental condition, in each culture. All experiments were performed in duplicate.

To identify functional subpopulations of beta cells and to determine whether these subpopulations were differentially affected by the experimental treatments, a frequency distribution of immunoplaque areas was constructed with data pooled from three different experiments by duplicate. According to the presence of different secreting subpopulations (24), we considered two classifications: small plaque-forming cells (SP: area of immunoplaques <1,900 µm^2^) and large plaque-forming cells (LP: area >1,900 µm^2^) (25).

### Statistical Analysis

All data are reported as the mean ± SEM; where “*n*” denotes the number of cells or experiments. Statistical significance is reported at the 95% confidence interval and was obtained with the one-way analysis of variance (Statistic, version 7.0).

## Results

### Detection of T-Type Current in Neonatal, P20, and Adult Beta Cells

**Figure 1:**
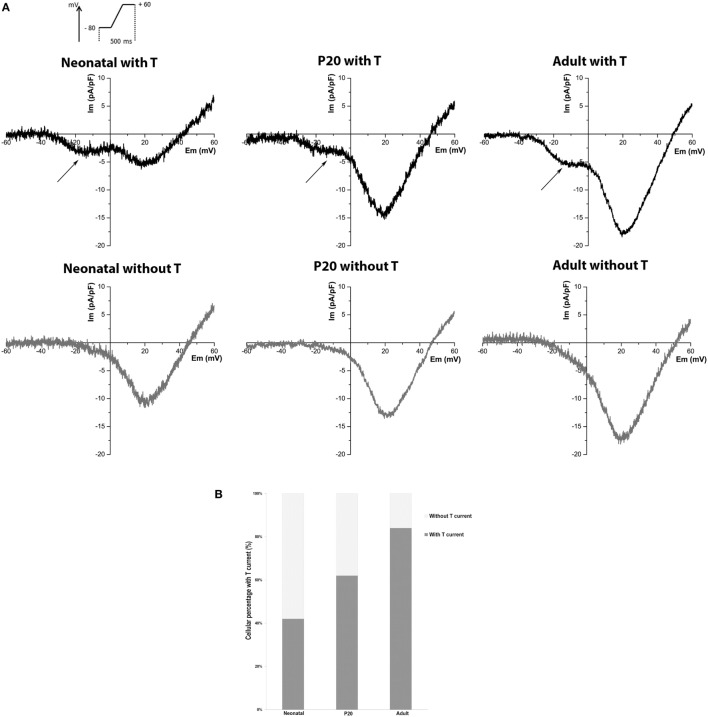
Detection of T-type current in neonatal, P20, and adult beta cells. **(A)** Representative recordings of global calcium currents observed in neonatal, P20, and adult beta cells. Arrows represent T-type calcium current. **(B)** Quantification of the beta-cell percentage with and without T-type calcium current in neonatal (*n* = 76), P20 (*n* = 78), and adult (*n* = 86) beta cells.

We recorded LVA and HVA Ca^2+^ currents from neonatal, P20, and adult individual beta cells with the whole-cell configuration of the patch-clamp technique, using Ca^2+^ as a charge carrier. As T-type channels have not been studied in detail in early stages of development, we were interested in knowing the percentage of beta cells where this current is detectable. We consider a threshold >2 SD of noise at −10 mV (peak of LVA current). Figure 1A shows representative records of neonatal, P20, and adult beta cells with or without T-type current and the percentages of detection (Figure 1B). LVA current was detected in 42% of neonatal cells (*n* = 76), 62% of P20 (*n* = 78), and 84% of adult cells (*n* = 86). Interestingly, the percentage of detection of T-type current increased with the maturation of beta cell.

### Biophysical Properties of LVA and HVA Calcium Currents in Neonatal, P20, and Adult Beta Cells

**Figure 2:**
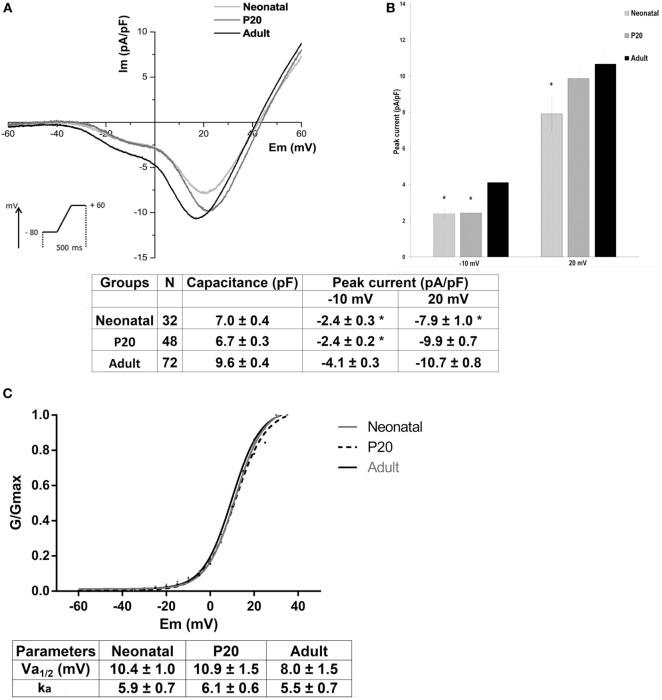
Biophysical properties of low-voltage-activated (LVA) and high-voltage-activated (HVA) calcium currents in neonatal, P20, and adult beta cells. **(A)** Ca^2+^ current density (Im, pA/pF) and voltage relationship (IV) of neonatal (black line, *n* = 32), P20 (red line, *n* = 48), and adult cells (blue line, *n* = 72). Protocol: voltage ramp from −80 to +60 mV, 500 ms duration, and 0.5 mV/ms slope, HP = −80 mV. **(B)** Quantification of capacitance cell and peak current density at −10 and +20 mV corresponding to LVA and HVA currents, respectively, in neonatal, P20, and adult beta cells. Data are expressed as current density average ± SEM, *n* denotes the number of cells recorded. **(C)** Voltage activation curves of HVA channels, which represent the Boltzmann fit to the data obtained from a protocol of depolarizing test pulses from −60 to +40 with 5 mV increments (top panel) and comparison of Boltzmann parameters calculated for plots of Ca^2+^ conductance in neonatal, P20, and adult beta cells (bottom panel). Data are mean ± SEM of 9 neonatal, 15 P20, and 9 adult cells. *p ≤ 0.05 denotes significant differences with respect to adult cells, Tukey test (analysis of variance).

To determine the biophysical properties of LVA and HVA calcium currents in beta cells, we analyzed only cells with the T-type current. Figure 2A shows the mean current-voltage relationships of ICa^2+^ in neonatal, P20, and adult beta cells. In all ages, inward currents activated at nearly −40 mV and reached a maximum of LVA current around −10 mV and a maximum of HVA current around +20 mV during ramp protocol. Adult cells presented the higher calcium currents, and specifically HVA current amplitude increased with maturation stages. Adult beta cells increased T-type calcium current density by 41% compared with neonatal and P20 cells whereas they increased HVA current density by 26% compared with neonatal cells (Figure 2B).

Figure 2C shows voltage activation curves of HVA channels in neonatal, P20, and adult beta cells. These curves represent the Boltzmann fit to the data obtained from a protocol of depolarizing test pulses from −60 to +40 with 5 mV increments (see Materials and Methods). No differences were found in voltage dependence, half-maximal activation (Va_1/2_), or steepness factor (ka) of activation curves between ages.

### Variability of HVA Calcium Currents in P20 Beta Cells

Due to the importance of P20 in the maturation process in beta cells, where previous studies in our group have shown the presence of hyperinsulinemia and insulin resistance in these rats, we analyzed the behavior of calcium currents at this maturation stage.

**Figure 3:**
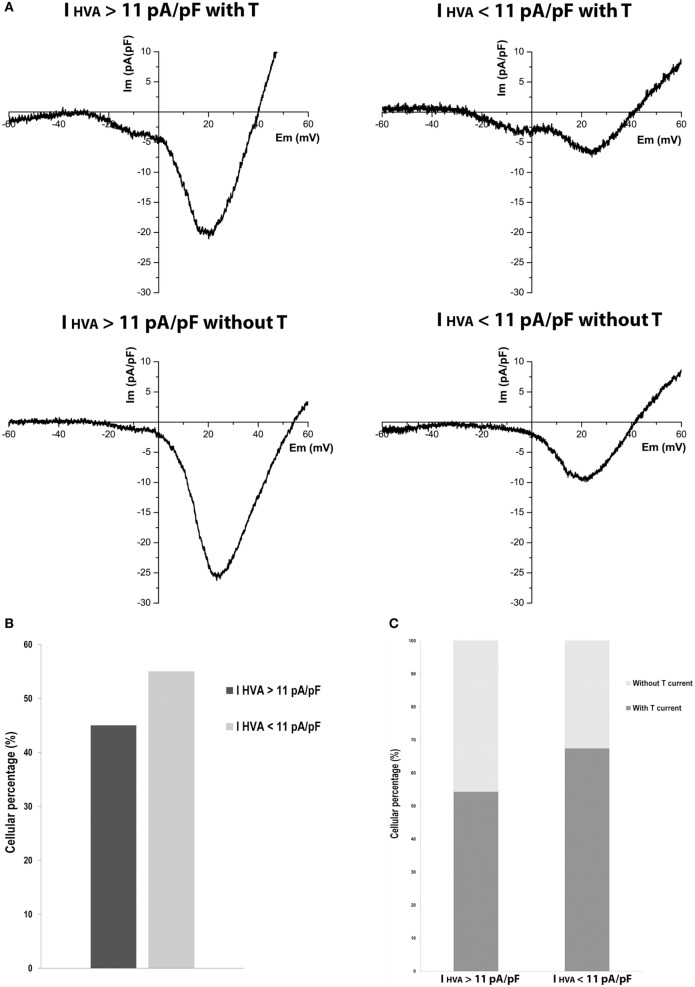
Variability of high-voltage-activated (HVA) calcium currents in P20 beta cells. **(A)** Representative recordings of global calcium currents observed in P20 beta cells with HVA currents higher or less than HVA currents of adult beta cells with or without T-type current. Protocol: voltage ramp from −80 to +60 mV, 500 ms duration, and 0.5 mV/ms slope. **(B)** Quantification of the P20 beta cells percentage with HVA currents higher (*n* = 35) and less (*n* = 43) than HVA currents of adult beta cells. **(C)** Quantification of the P20 beta cells percentage represented in **(B)** with and without T-type calcium current.

Heterogeneous behavior in calcium currents was observed (Figure 3A). Curiously, we found cells with HVA currents much higher than mean HVA current density of adult cells, and this was independent of the presence of LVA current. Cells with HVA current density higher than 11 pA/pF were 45% (Figure 3B). Also, 54% of cells with HVA current density higher than 11 pA/pF and 67% of cells with HVA current density less than 11 pA/pF have T-type current (Figure 3C). This heterogeneity suggests the existence of beta-cell subpopulations at P20 regarding calcium currents.

### Differential Expression of T-Type Channels (α1G, α1H, and α1I Subunits) in Neonatal, P20, and Adult Pancreatic Beta Cells

**Figure 4:**
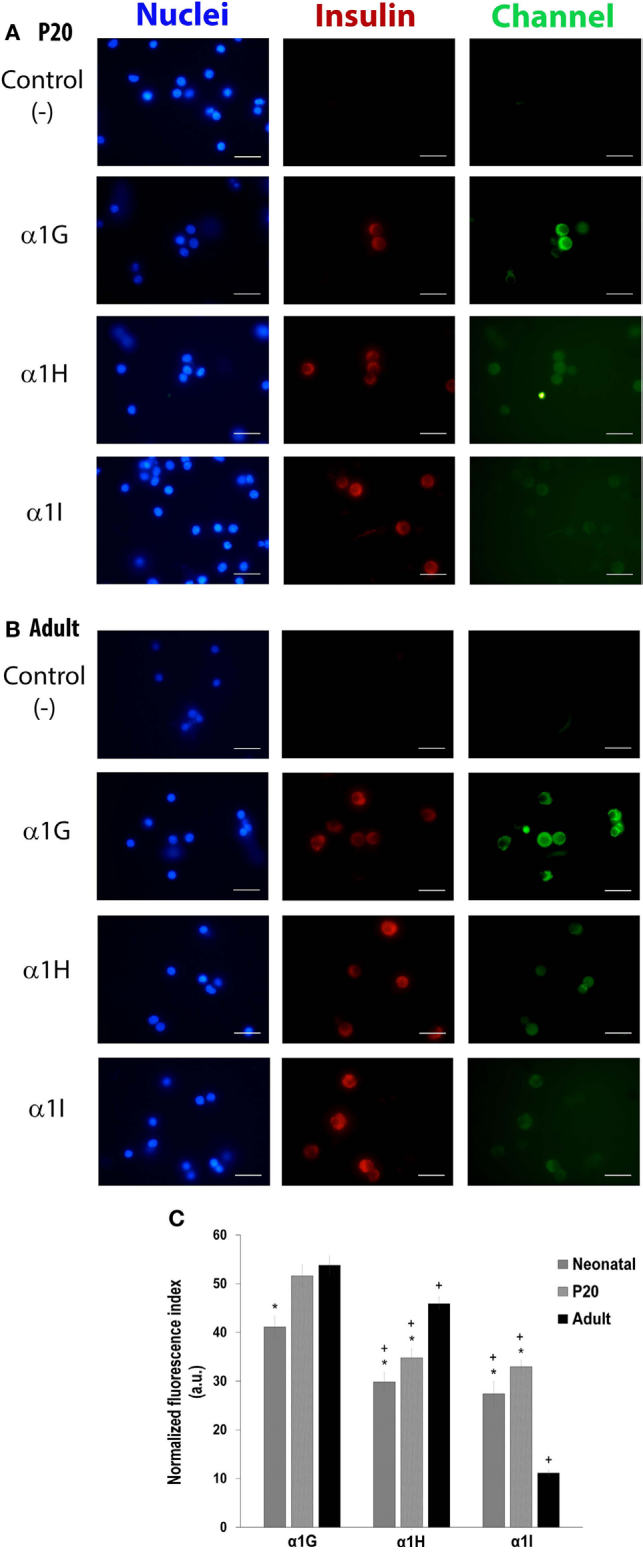
Differential expression of T-type channels (α1G, α1H, and α1I subunits) in neonatal, P20, and adult pancreatic beta cells. Representative fluorescence micrographs of expression of α1G, α1H, and α1I subunits of P20 **(A)** and adult **(B)** cells in culture, showing differences in the levels of immunostaining. Scale bars represent 20 µm. **(C)** Immunofluorescence index measured from about 150–200 cells per isoform per age. Symbols denote significant differences with respect to adult cells (*) and with respect to the α1G subunit in each age (+), *p* ≤ 0.05, Dunnet test (analysis of variance).

The most prominent Ca^2+^ current density recorded from adult beta cells could result from higher expression levels of the calcium channels. To assess this hypothesis, we studied the expression of LVA channels and determined if there is a differential expression of α1G, α1H, and α1I subunits in neonatal, P20, and adult beta cells. As shown in Figures 4A–C, α1G, α1H, and α1I subunits-specific immunostaining is observed in beta cells obtained from all stages in our study. However, regardless of the developmental stage, α1G subunit-specific fluorescence is more abundant than other subunits (Figures 4A–C). Also, α1G is expressed in all beta cells of three ages. The percentages of expression of α1H are 98, 96, and 99% in neonatal, P20, and adult beta cells, respectively, whereas α1I is the less expressed subunit with 94% of positive beta cells in the three ages (Table 1). Interestingly, α1G and α1H subunits-specific fluorescence in adult beta cells is more abundant than in neonatal and P20 cells (Figure 4C). However, α1I subunit-specific immunostaining in adult cells is less abundant than in cells of other ages. In general, the expression of different subunits of T-type calcium channels varies during the postnatal development of beta cells from rats.

**Table 1:** Percentage of beta cells expressing α1G, α1H, and α1I subunits of T-channels in neonatal, P20, and adult cells.

	Detection of T-type calcium channels in beta cells (%)		
Subunits	Neonatal	P20	Adult
α1G (Cav 3.1)	100	100	100
α1H (Cav 3.2)	98	96	99
α1l (Cav 3.3)	94	94	94

### Effect of Calcium Channel Inhibitors on LVA and HVA Calcium Currents in Adult Beta Cells

After determining the biophysical properties of the LVA and HVA calcium currents in beta cells, we evaluated the effects of calcium channel inhibitors on currents in adult beta cells. We used the classic inhibitor of L-type HVA channel nifedipine (5 µM) and a variety of inhibitors with different specificities on LVA channels: mibefradil (1 and 10 µM), NiCl_2_ (50 µM) and TTA-A_2_ (5 and 50 µM).

**Figure 5:**
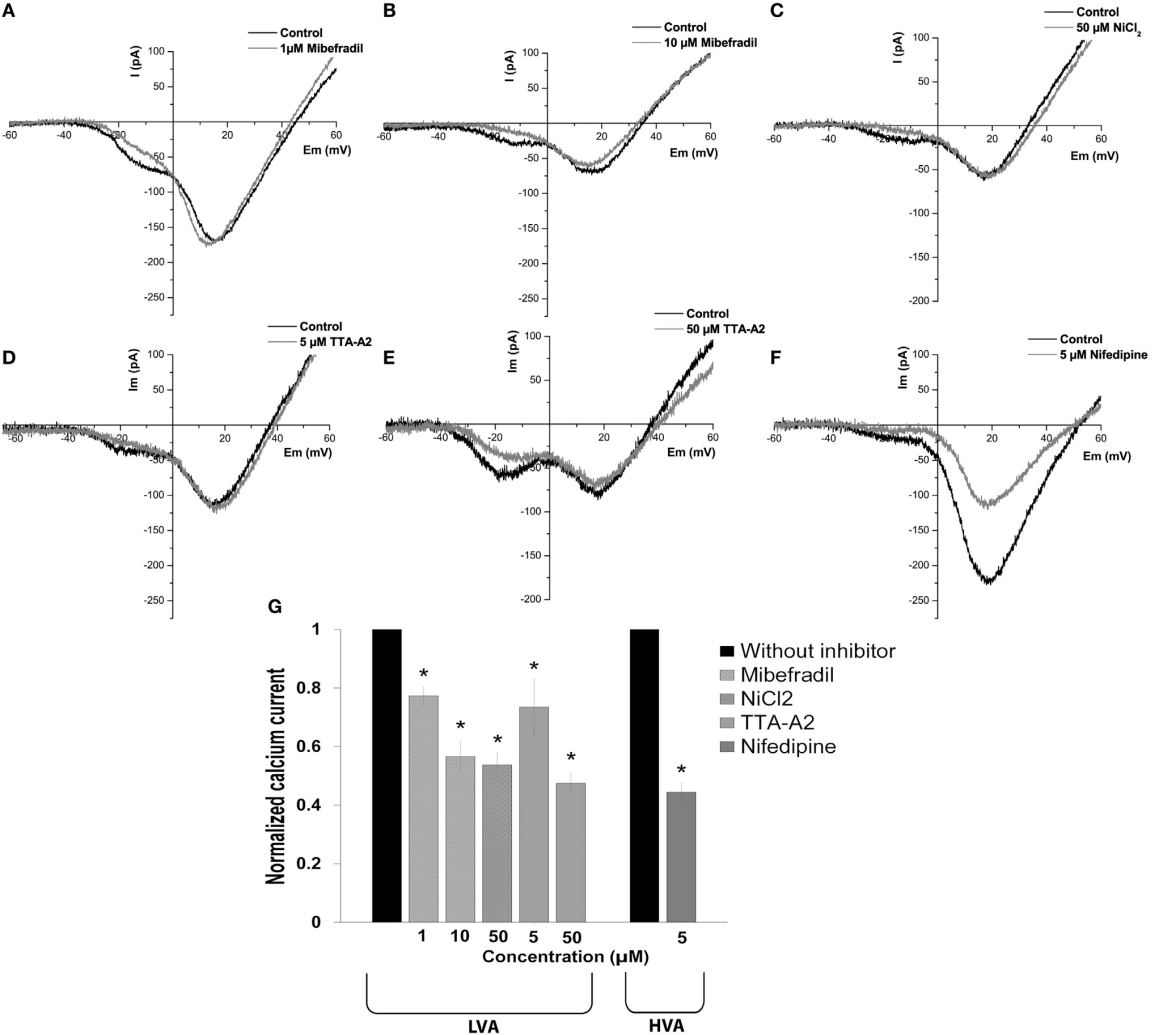
Effect of calcium channel inhibitors on low-voltage-activated (LVA) and high-voltage-activated (HVA) calcium currents in adult beta cells. **(A–F)** Representative recordings of global calcium currents observed in adult beta cells before and after of calcium channel inhibitors: mibefradil (1 and 10 µM), NiCl_2_ (50 µM), TTA-A_2_ (5 and 50 µM), and nifedipine (5 µM), respectively. Protocol: voltage ramp from −80 to +60 mV, 500 ms duration, and 0.5 mV/ms slope. **(G)** Normalized calcium current after of calcium channel inhibitors: 1 µM mibefradil (*n* = 5), 10 µM mibefradil (*n* = 6), NiCl_2_ (*n* = 6), 5 µM TTA-A_2_ (*n* = 5), 50 µM TTA-A_2_ (*n* = 3), and nifedipine (*n* = 6). *p ≤ 0.05 denotes significant differences compared with cells without inhibitors, paired *t*-test.

To determine the dose dependence of mibefradil block, we applied 1 and 10 µM concentrations. Representative current traces are shown in Figures 5A,B, before and after mibefradil at 1 and 10 µM, respectively. Mibefradil block was dose-dependent with 23 and 44% of the T-type current block at 1 and 10 µM, respectively (Figure 5G).

The sensitivity of native HVA and T-type currents to NiCl_2_ is variable in neurons, with αIH as the most sensitive subunit with an IC_50_ in the micro molar range (26). Figure 5C shows representative calcium current traces before and after the application of 50 µM NiCl_2_. The NiCl_2_ block was selective for T-type current with 46% of decrease of the peak current (Figure 5G).

TTA-A_2_ also selectively blocked the LVA current, and the block was concentration dependent (Figures 5D,E) with 27 and 53% of current block at 5 and 50 µM, respectively (Figure 5G). Nifedipine (5 µM) blocked the HVA current by 56% (Figures 5F,G).

### Effect of Calcium Channel Inhibitors on Basal and Glucose-Induced Insulin Secretion in Adult Beta Cells

To evaluate the role of HVA and LVA channels in insulin secretion by single cells, we measured the effect of the inhibitors previously assessed in our electrophysiological studies on basal and glucose-induced insulin secretion by RHPA.

Curiously, only mibefradil and TTA-A_2_, specific inhibitors of T-type calcium channels, decreased the percentage of plaque-forming cells (F) in 5.6 mM glucose. In the same basal condition, nifedipine and TTA-A_2_ reduced the plaque area. However, all drugs decreased the percentage of insulin-secreting cells and the plaque area in 15.6 mM glucose (Table 2).

**Table 2:** Insulin secretion by single beta cells in response to a 1 h incubation to blocker drugs in different extracellular glucose concentrations.

Treatment	Glucose (mmol/L)	Percentage of plaque-forming cells	Plaque area (μm^2^)
Control	5.6	23.9 ± 0.9	1,291 ± 258
	15.6	62.2 ± 2.0	2,200 ± 248
Nifedipine (5 µM)	5.6	21.4 ± 6.8	994 ± 156^+^
	15.6	23.6 ± 4.1*	917 ± 117*
Mibefradil (1 µM)	5.6	17.3 ± 3.7^+^	1,088 ± 63
	15.6	24.8 ± 5.4*	1,055 ± 123*
Ni CI_2_ (50 µM)	5.6	24.3 ± 9.5	1,250 ± 284
	15.6	42.6 ± 3.1*	1,516 ± 114*
TTA-A2 (50 µM)	5.6	13.3 ± 0.9^+^	883 ± 19^+^
	15.6	41.1 ± 0.2*	1,289 ± 268*
Nifedipine + TTA-A2	5.6	7.8 ± 0.7^+^	497 ± 95^+^
	15.6	17.4 ± 4.8*	581 ± 56*

Symbols, (+) denotes significant differences with respect to control cells at 5.6 mM glucose, (*) denotes significant differences with respect to control cells at 15.6 mM glucose Dunnet test (analysis of variance).

**Figure 6:**
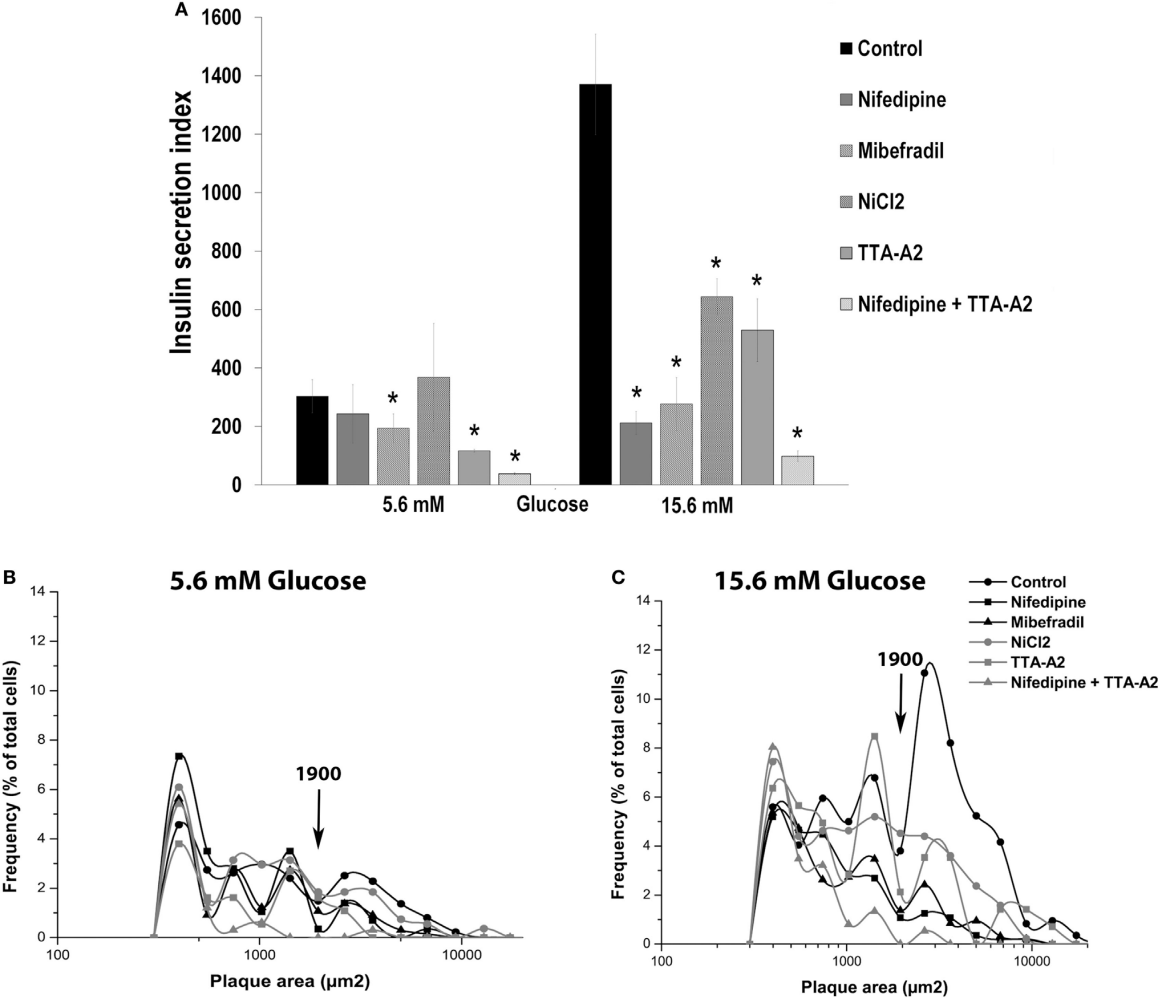
Effect of calcium channel inhibitors on basal and glucose-induced insulin secretion in adult beta cells. **(A)** Overall insulin secretory activity (insulin secretion index) of adult beta cells incubated with nifedipine (5 µM), mibefradil (1 µM), NiCl_2_ (50 µM) and TTA-A_2_ (50 µM), exposed at basal (5.6 mM glucose), and stimulating (15.6 mM glucose) conditions. Bars represent the mean ± SEM of three different experiments by duplicate. *p ≤ 0.05 denotes significant differences with respect to control cells at 5.6 and 15.5 mM glucose. Dunnet test (analysis of variance). **(B,C)** Frequency distribution of functional subpopulations of beta cells incubated with inhibitors of **(A)** observed in basal **(B)** and stimulating **(C)** glucose conditions.

Figure 6A shows the overall secretory activity of beta cells, expressed as insulin secretion index. Similar to the percentage of plaque-forming cells, only mibefradil and TTA-A_2_ reduced the insulin secretion index in the basal conditions. TTA-A_2_ decreased the secretion index more than mibefradil, and the reduction of secretion was near to 60%. However, all drugs were effective in decrease the secretion index in stimulating conditions. Nifedipine and mibefradil were the most effective in 15.6 mM glucose, with 85 and 80% of the reduction in the amount of insulin secreted by single cells, respectively. The combination of nifedipine and TTA-A_2_ drastically decreased the percentage of plaque-forming cells, the plaque area, and the insulin secretion index both in basal and stimulating glucose conditions (Table 2; Figure 6A).

It is interesting to note that all treatments drastically reduced the percentage of LP cells (plaque area > 1,900 μm^2^) in 15.6 mM glucose, which represent the beta-cell subpopulation with the highest rate of insulin secretion. These cells are responsible for most of the insulin secreted by a beta-cell culture (Figure 6C). This effect was less marked in 5.6 mM glucose (Figure 6B). While nifedipine decreased almost wholly the LP subpopulation in stimulating conditions, TTA-A_2_ had the same impact on basal conditions (Figures 6B,C). The fraction of SP cells (plaque area < 1,900μm^2^), however, was similar in all the experimental groups in 15.6 mM glucose (Figure 6C). The combination of nifedipine and TTA-A_2_ abolished almost entirely the subpopulation of LP cells both in basal and stimulating glucose conditions.

These results confirm that calcium currents (HVA and LVA) contribute in an important way to the insulin secretion, both in basal and stimulating conditions. While it is well accepted that L-type calcium channels are the most important calcium pathway in GSIS, our results also suggest that T-type calcium channels contribute to both, basal and stimulating insulin secretion.

## Discussion

Pancreatic beta cell acquires functional maturity during the first month of life, allowing them to secrete robust amounts of insulin in response to increases in extracellular glucose concentration.

It has been well established that fetal islets poorly respond to glucose stimulation with insulin secretion. In one study using perfused rat pancreas at 3 days before birth showed the absence of biphasic response to 16.7 mM glucose (27). They attributed this immaturity to failure in the metabolism of nutrients to couple with the K_ATP_ channel and thus preventing the depolarization of the beta-cell membrane.

An impaired glucose metabolism in beta cells from 21 days fetal rats was also reported (28). For these authors, this immature glucose metabolism results in impaired regulation of the K_ATP_, suggesting an altered mitochondrial function, which leads to a defective depolarization, no increase in the free intracellular calcium, and no insulin secretion. However, other stimulators of insulin release distinct to glucose, for example, leucine, arginine, and theophylline, promote insulin secretion at this fetal stage, suggesting that at least a part of the secretory machinery is developed (29). This study also mentions that pancreatic islets of fetal rats are not able to respond with calcium net uptake with various initiators of insulin release, which cause depolarization and insulin release in adult islets of rats; suggesting that stimulation of calcium uptake *via* the VGCC is not possible in the fetal state.

In contrast, other study showed that various secretagogues (glyceraldehyde, leucine, and arginine) except glucose were able to stimulate fetal islets and to increase the intracellular calcium (30). These authors attributed the immature insulin secretory response to glucose to the inability of the fetal beta cells to translate glucose stimulation into the increase in [Ca^2+^]i necessary for insulin exocytosis, the defect that can be at the level of the glucose transporter, or in the linkage between glycolysis and mitochondrial oxidation.

A posterior study in fetal porcine and human islet-like cell clusters revealed that glucose and leucine failed to elicit an increase in [Ca^2+^]i, and suggested that the differences in the effects of secretagogues are attributable to the time-dependent maturation of beta cells (31).

The different gestational times in the studies could justify the controversy about the mechanisms for explaining the immaturity in insulin secretion and glucose response in early stages of development. In addition to the secretagogues, other molecules may have a role in the differentiation and maturation of pancreatic beta cells. For example, PDZ-domain containing-2 peptide, which promoted the expression of calcium channels in differentiated islet-like cell clusters, triggering calcium influx under KCl stimulation, and drives their maturation and the ability to secrete insulin (32).

While during the fetal stage, the homeostasis of glucose is dependent on the mother, birth is a critical developmental window due to the nutrition of organisms becomes utterly dependent on food availability. Neonatal beta cells also are considered immature. However, adult beta cells can detect changes in the extracellular concentration of glucose and they are considered mature. At this stage, insulin secretion is biphasic, with a transient first fast phase and a second sustained phase of secretion (1). The need to maintain the glycemia could be a driving force for beta cells to mature and secrete insulin in response to glucose concentration. We think that the change in animal feed can lead to modifications in the response machinery of beta cells, including changes in ion channels activity.

To our knowledge, this is the first work that analyzes the biophysical properties and expression pattern of voltage-dependent calcium channels, comparing three stages of postnatal development of rat pancreatic beta cells.

It is well known that calcium channels are very important for insulin secretion in beta cells. It is well accepted that HVA channels, individually type L channels, are responsible for the increase in intracellular calcium concentration and insulin exocytosis (10). However, LVA currents have been poorly studied.

Interestingly, our results showed that the percentage of detection of T-type current increased with the maturation of beta cell, doubling from neonatal to adult cells. Curiously, other studies related to the expression of T-type channels during development in different cell types have shown an inverse behavior. For example, T-type calcium channels are expressed in the fetal heart but then disappear from ventricular myocytes after birth (33). They observed that 35% of myocytes expressed functional T-type channels at birth, similar to our results (42%), however, this percentage decreases during the first week of life until by the end of the first week and no myocytes had functional T-type channels. Curiously, a study suggested the absence of T-type channels in human fetal pancreatic beta cells (31).

A similar behavior to ventricular myocytes was observed in some neuronal and skeletal muscle cells, in which T-type channels are highly expressed at the embryonic and perinatal period and disappear during the adult stage (34). Our findings also are distinct to the reported in other hormone-secreting cells like chromaffin cells, in which low-threshold Ca^2+^ currents can be hardly detected in adult cells of mammalian species because LVA channels are either absent or the expression is not robust in these cells [reviewed by Ref. (35)]. In the case of beta cells and insulinoma lines, the T-type current had been observed in adult rats and human beta cells, INS-1 and RINm5F, but not in mouse beta cells (36). We detected calcium currents mediated by HVA channels in all cells during development of beta cell. Other studies have indicated that even from the fetal stage, beta cells have functional HVA channels (28, 30, 31).

As we have mentioned, the entry of calcium through the L-type channels is determinant of the intracellular calcium concentration that triggers the exocytosis of insulin. However, the calcium-releasing channels present in the endoplasmic reticulum (ER) of beta cells also have an important contribution to intracellular calcium, and they can modulate the Ca^2+^ signaling induced by depolarization (37, 38). The leading families of intracellular Ca channels are the ryanodine receptors (RyR) and the inositol 1,4,5-trisphosphate receptors (IP3Rs). They can sequester Ca^2+^ that enters through the VGGCs, or they can contribute to the release of Ca^2+^ from the intracellular stores (38). Opposed to cardiomyocytes and other cell types, the functional role of Ca^2+^ release from the ER in response to glucose is somewhat controversial. However, an increase in the expression of IP3R1 and RyR2 in pancreatic beta cells has been reported [reviewed by Ref. (39)]. Several studies have indicated that these intracellular channels play an essential role in the regulation of insulin secretion. For example, mice defective in the expression of IP3R have an inadequate release of insulin, and the Ca^2+^-induced Ca^2+^ release *via* IP3R is involved in the mechanisms by which cAMP amplifies insulin secretion. Also, the potential participation of the RyR2 channels in insulin secretion was suggested by the potentiation of Ca^2+^ response after caffeine treatment [reviewed by Ref. (39)].

A recent study showed that sirolimus, an immunosuppressive drug, depletes calcium from ER, and decreases glucose-stimulated mitochondrial Ca^2+^ uptake, which could lead to a reduction in GSIS in human and murine islets and INS-1 cells (40). Interestingly, both IP3Rs and RyRs are involved in ER stress in pancreatic beta cells [reviewed by Ref. (39)]. RyR2 dysfunction in beta cells leads to ER stress and mitochondrial dysfunction, which can cause oxidative stress, bioenergetic deficit, and consequently impaired insulin release (37). In future studies, it would be interesting to investigate the presence of intracellular calcium release channels and its functional role during development of rat beta cells.

We also demonstrated by immunofluorescence the presence of the three T-type calcium channel isoforms in neonatal, P20, and adult beta cells of rats. Although many studies have not reported the presence of α1I subunit (Cav 3.3) in some cell types, other authors, like us, also have observed the expression of the three T-type channel isoforms, for example, in human and mouse spermatogenic cells (41). Nevertheless, we observed that the expression patterns differ during development and between the isoforms at the same age. We showed that α1G subunit (Cav 3.1) is the isoform most expressed in all stages and its expression is highest in P20 and adult cells than in neonatal cells. A similar result was observed between neonatal and adult beta cells for this isoform (18). Studies in neuroendocrine cells like chromaffin cells also have shown that Cav 3.1 is the T-type channel isoform most expressed [reviewed by Ref. (35)]. Cav 3.1 isoform has been detected in beta cells of rats and INS-1 cells, but not in beta cells of mouse (36).

Our results showed that, distinct to other isoforms, Cav3.2 expression progressively increased from one stage of maturation to the other. This confirms the validation approach of our group, whose microarray analysis revealed a higher expression of different calcium channel isoforms, like α1H subunit (Cav3.2), in adult beta cells compared with P20 cells (20). Neurons and myocytes also exhibit a variable pattern of calcium channel expression during different stages of development (42–44).

The highest expression of Cav 3.1 and Cav 3.2 in adult beta cells could be related to a more significant T-type calcium current density at this stage. Conclusively, our data showed that T-type calcium current density in adult beta cells is higher than in neonatal and P20 cells. These results suggest that the high level of expression of T-type calcium channel isoforms, particularly Cav 3.1 and Cav 3.2, may lead to the increased T-type calcium current density that we observed at this stage of development. However, it would be interesting to be more studies that allow ratifying this correlation, due to our electrophysiological records were performed in primary cultures and it very difficult to discriminate between isoforms.

Interestingly, our data also show that HVA calcium current density increases with the stage of development of beta cell. Similarly, in neonatal cells, barium current density is lower than in adult cells (18).

These results about the differential expression pattern of calcium channels isoforms and changing current densities during development are very interesting because it could be related to the functional maturation of rat pancreatic beta cells.

A crucial moment in development is near the weaning time (20 days), where our lab was the first to propose the weaning period as a critical maturation window of pancreatic development. At P20, animals show physiological insulin resistance, and this could be due to the change from a lipid-rich milk diet to another high in carbohydrates, which represents a metabolic challenge, and it plays an essential role in GSIS (3). Taking into account these previous observations, we were interested in studying the calcium currents at this stage. Surprisingly, we found a heterogeneous behavior related to calcium currents within the beta-cell population. As we discussed earlier, mean HVA calcium current density increase with the maturation of beta cell. However, we observed that about 45% of beta cells at P20 have HVA current densities much higher than in adult cells. We also found that this hyperactivity in HVA channels is independent of the presence of T-type current.

This study represents the first description of the calcium currents in P20 cells of rats. It is interesting to note the importance of this period in the maturation process of beta cells. Our present findings may contribute to explain an interesting physiological state of hyperglycemia and hyperinsulinemia detected at this period of development in rats (3). Indeed, further studies are necessary to explore the association between exacerbated calcium currents and the hyperinsulinemia observed.

We also demonstrated that blockers of calcium channels subtypes T and L can partially inhibit insulin secretion in basal and stimulated conditions of glucose in adult beta cells. We found that 53% of the LVA-calcium current and near to 60% of HVA-calcium current in adult beta cells are TTA-A_2_ and nifedipine sensitive, respectively. Our results are in accordance with previous observations in our lab for the block of barium currents (18, 25). In order to explore the contribution of both channels types to insulin secretion, we evaluated the effects of different blockers on insulin secretion by RHPA.

An advantage of the RHPA is that secreting beta cells can be unambiguously identified. Previous studies have shown that the RHPA is a useful technique for identifying and studying secretion from individual pancreatic beta cells (23). We have shown that the calcium channels in rat beta cells are functionally very important for stimulus-secretion coupling. The RHPA showed that mibefradil (36%) and TTA-A_2_ (61%) had significant inhibitory effects on insulin secretion at 5.6 mM glucose in adult beta cells, which is near the threshold for activating electrical activity (45). Our data support the hypothesis that T-type calcium channels are involved in the process of insulin release in basal conditions of glucose. Others have also shown that the inhibition of T-type calcium channels reduced insulin secretion by 60–70% at 6 mM glucose in human pancreatic beta cells (46). Other authors have proposed that T-type Ca^2+^ channels may increase the frequency of the spontaneous [Ca^2+^]i spikes, resulting in premature release of insulin in non-stimulatory glucose conditions (47).

Our data showed that all drugs clearly inhibited insulin secretion under stimulatory glucose conditions. Between them, nifedipine (85%) almost completely blocked secretion index at 15.6 mM glucose without affecting basal insulin secretion. These results confirm the functional importance of HVA calcium channels in beta cells, the principal responsible of insulin exocytosis. Our data are in agreement with others studies of our group where previously we observed that, in adult beta cells, nifedipine completely blocked GSIS without affecting basal secretion (25). Other studies have also shown that blockade of L-type calcium channels inhibited GSIS in rat beta cells (4) and human beta cells (13, 46). Our data showed that blockade of T-type calcium channels also inhibited insulin secretion at 15.6 mM glucose. The effect of these drugs suggests that T-type calcium channels are also important for insulin secretion in stimulating conditions. Another study of our lab showed that pimozide, another T calcium channels blocker, but less selectively than mibefradil, inhibited insulin secretion by 58% at 15.6 mM glucose (4). Others have also shown that the blockage with NiCl_2_ decreases insulin secretion in INS-1 cells (17).

The Ca^2+^ channel blockers tested here affect the RHPA in two ways: (1) by decreasing the percentage of plaque-forming cells (insulin-secreting cells), which suggests that the blockers prevent the recruitment of previously silent cells to the secretory activity (24) and (2) by decreasing the immunoplaque area, which is proportional to the amount of hormone secreted by individual cells.

It is well known that insulin secretion by single beta cells is heterogeneous and it has been shown that there are functional subpopulations of adult rat beta cells (24, 48, 49). One subpopulation secretes more insulin (LP cells) than the other (SP cells), but, the LP cells have the highest secretion rate and are responsible for nearly 75% of the total secretion (24). In our study, the beta-cell subpopulation most affected by all calcium channels blockers was the LP cells, because these cells are decreased in both basal and stimulating glucose conditions. Interestingly, the effects of TTA-A_2_ and nifedipine were the most evident at 5.6 and 15.6 mM glucose, respectively. These results imply that a beta-cell subpopulation is preferentially modulable by calcium channels blockers, suggesting that different cell types could have variable calcium channel density and/or secretory machinery.

The main results of this study are summarized as follows (1) the percentage of detection of T-type current increases with the stage of development of beta cell. (2) T-type calcium currents in adult cells are higher than in neonatal and P20 beta cells. (3) HVA current density increases with the maturation of beta cell. (4) Calcium currents are heterogeneous at P20. Almost half of the cells have HVA current densities higher than in adult cells, and it is independent of the presence of T-type current. (5) Immunocytochemistry revealed the presence of α1G, α1H, and α1I subunits of LVA channels at all ages. The Cav 3.1 subunit was the most expressed. Adult beta cells expressed more Cav 3.1 and Cav 3.2 than neonatal and P20 cells. (6) Mibefradil and TTA-A_2_ significant inhibit insulin secretion at 5.6 mM glucose, which supports the idea of the physiological importance of T-type channels at basal glucose conditions. (7) Calcium channels blockers, particularly nifedipine and TTA-A_2_, drastically decrease the LP beta-cell subpopulation in both basal and stimulating glucose conditions.

Taken together, we conclude that LVA and HVA calcium channels exhibit a variable pattern of expression and electrical activity during development of pancreatic beta cells of rats, which may play essential roles during the physiological maturation of beta cells. We consider that our work brings new points of view on the study of the ontogeny of beta pancreatic cells, as well as the biophysics of the ion channels participating in insulin secretion.

## Ethics Statement

All methods used in this study were approved by the Animal Care Committee of the Instituto de Fisiología Celular, Universidad Nacional Autónoma de México. Animal care was performed according to the International Guiding Principles for Biomedical Research Involving Animals, Council for International Organizations of Medical Sciences, 2010.

## Author Contributions

MH and NG-D planning the study, MH, NG-D, and MV designing experiments, MH and NG-D obtaining grants, NG-D, MV, CD-G, and CS-S perform experiments, MH, NG-D, MV, CD-G, and CS-S analyzing data, MH, NG-D, MV, CD-G, and CS-S writing and reviewing the manuscript.

## Conflict of Interest Statement

The authors declare that the research was conducted in the absence of any commercial or financial relationships that could be construed as a potential conflict of interest.

## Supplementary Material

Figure S1 Biophysical properties of low-voltage-activated (LVA) and high-voltage-activated calcium currents in neonatal, P20, and adult beta cells. Ca^2+^ current density (Im, pA/pF) and voltage relationship (IV) of neonatal (*n* = 11), P20 (*n* = 34), and adult cells (*n* = 8). Protocol: depolarizing test pulses from -60 to +40 with 5 mV increments, HP = -80 mV. Quantification of peak current density at -10 and +20 mV corresponding to LVA and HVA currents, respectively, in neonatal, P20, and adult beta cells. Data are expressed as current density average ± SEM; n denotes the number of cells recorded. **p* ≤ 0.05 denotes significant differences with respect to adult cells, Tukey test (ANOVA).

Figure S2 Representative images of RHPA of adult beta cells exposed at 5.6 and 15.6 mM glucose in each condition, controls and incubated with nifedipine (5 µM), mibefradil (1 µM), NiCl_2_ (50 µM), TTA-A2 (50 µM) and the combination of nifedipine + TTA-A2.
