# Supplementary figures and images for: Calcium Channels in Postnatal Development of Rat Pancreatic Beta Cells and Their Role in Insulin Secretion
Source: Front Endocrinol (Lausanne). 2018 Mar 5;9:40. doi: 10.3389/fendo.2018.00040 (PMC5845110; doi:10.3389/fendo.2018.00040)

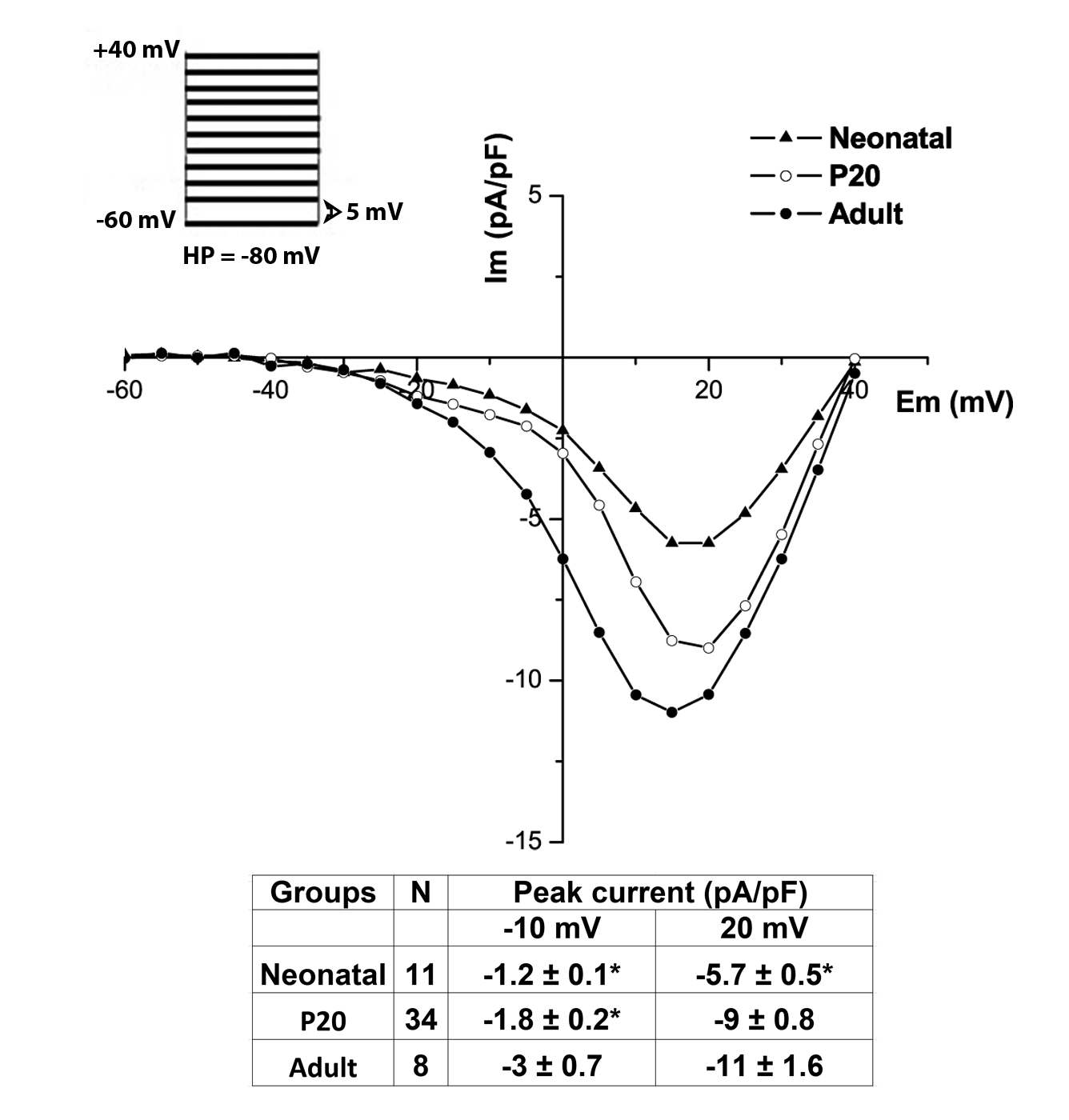

Supplement: Figure S1 — Biophysical properties of low-voltage-activated (LVA) and high-voltage-activated calcium currents in neonatal, P20, and adult beta cells. Ca2+ current density (Im, pA/pF) and voltage relationship (IV) of neonatal (n = 11), P20 (n = 34), and adult cells (n = 8). Protocol: depolarizing test pulses from -60 to +40 with 5 mV increments, HP = -80 mV. Quantification of peak current density at -10 and +20 mV corresponding to LVA and HVA currents, respectively, in neonatal, P20, and adult beta cells. Data are expressed as current density average ± SEM; n denotes the number of cells recorded. *p ≤ 0.05 denotes significant differences with respect to adult cells, Tukey test (ANOVA). [file Image_1.tif]

# Glucose

5.6 mM

15.6 mM

**Control**

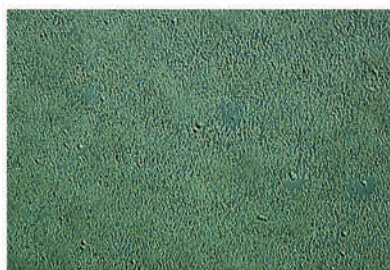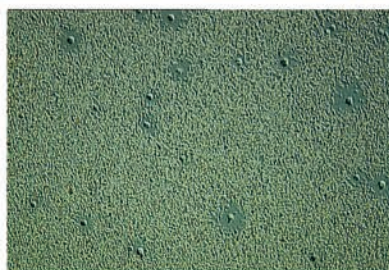

**Nifedipine**

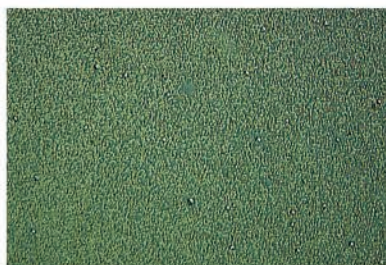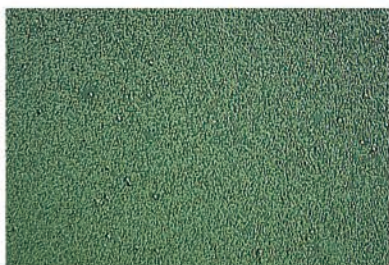

**Mibefradil**

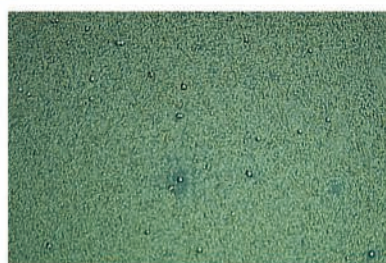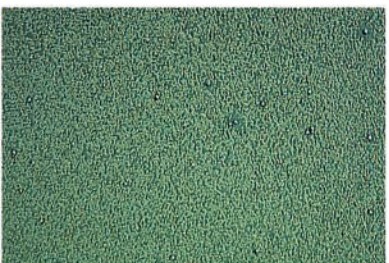

**NiCl<sub>2</sub>**

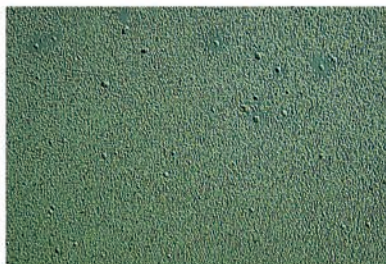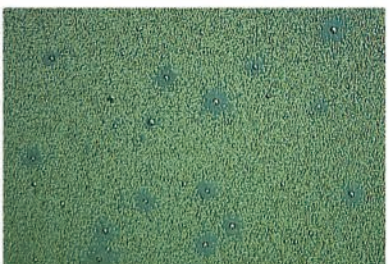

**TTA-A2**

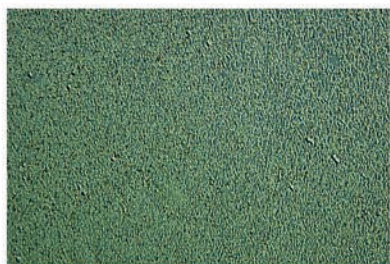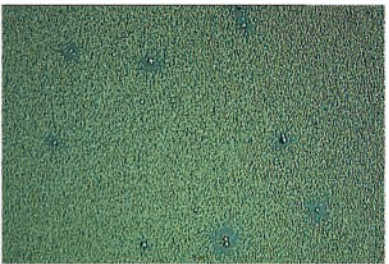

**Nifedipine  
+  
TTA-A2**

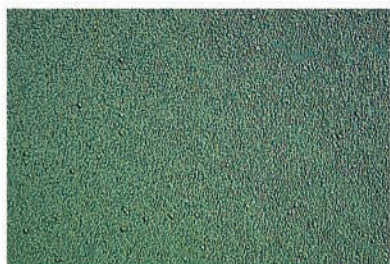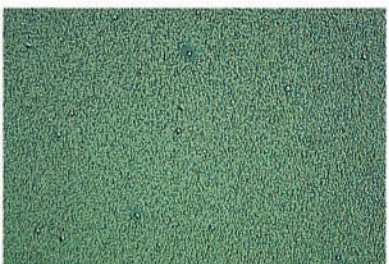

Supplement: Figure S2 — Representative images of RHPA of adult beta cells exposed at 5.6 and 15.6 mM glucose in each condition, controls and incubated with nifedipine (5 µM), mibefradil (1 µM), NiCl2 (50 µM), TTA-A2 (50 µM) and the combination of nifedipine + TTA-A2. [file Image_2.PDF]
